# Clinical application of multi-material artifact reduction (MMAR) technique in Revolution CT to reduce metallic dental artifacts

**DOI:** 10.1186/s13244-020-0836-1

**Published:** 2020-03-06

**Authors:** Yijuan Wei, Fei Jia, Ping Hou, Kaiji Zha, Shi Pu, Jianbo Gao

**Affiliations:** 1 Department of Radiology, the First Affiliated Hospital of Zhengzhou University, Zhengzhou, 450000 Henan China; 2 Department of Radiation Oncology, the First Affiliated Hospital of Zhengzhou University, Zhengzhou, 450000 Henan China

**Keywords:** Multi-material artifact reduction, CT, Dental artifact, Metal

## Abstract

**Background:**

This study aimed to explore the performance of Revolution CT virtual monoenergetic images (VMI) combined with the multi-material artifact reduction (MMAR) technique in reducing metal artifacts in oral and maxillofacial imaging.

**Results:**

There were significant differences in image quality scores between VMI + MMAR images and VMI+MARS (multiple artifact reduction system) images at each monochromatic energy level (*p* = 0.000). Compared with the MARS technology, the MMAR technology further reduced metal artifacts and improved the image quality. At VMI_90 keV_ and VMI_110 keV_, the SD, CNR, and AI in the Revolution CT group were significantly lower than in the Discovery CT, but no significant differences in these parameters were found between two groups at VMI_50 keV_, VMI_70 keV_, and VMI_130 keV_ (*p* > 0.05). The attenuation was comparable between two groups at any energy level (*p* > 0.05).

**Conclusions:**

Compared with the MARS reconstruction technique of Discovery CT, the MMAR technique of Revolution CT is better to reduce the artifacts of dental implants in oral and maxillofacial imaging, which improves the image quality and the diagnostic value of surrounding soft tissues.

## Key points

- Compared to the 64-slice Discovery CT VMI + MARS technique for image reconstruction, 256-slice Revolution CT VMI + MMAR technique for image reconstruction is better to reduce metal artifacts and background SD.
- The combined use of VMI_110 keV_ + MMAR technique is helpful for the observation and evaluation of small structures around the metal implants.
- The combined use of VMI_110 keV_ + MMAR technique also provides a better diagnostic tool in clinical practice.

## Background

With the improvement of living standards and the development of oral medical care, dental restoration and implantation of dentures become more common. On CT scanning of the head and neck and maxillofacial region, the dental fillings or implants (e.g., amalgam, cobalt-chromium, nickel-chromium, gold alloy) will inevitably cause strip-like artifacts, such as beam hardening and photon starvation artifacts, which significantly degrade the image quality of the oral cavity and buccal area, making it difficult to delineate important anatomical structures or pathologic conditions [1, 2]. The extensive clinical application of dual-energy CT (DECT) is an effective way for postoperative follow-up and evaluation of therapeutic efficacy of metal implants, such as hip prosthesis, spinal fixation rods, and dental fillings [3–6]. Some studies have confirmed the efficacy of virtual monoenergetic images [6–8] and multiple artifact reduction system (MARS) [9] in reducing metal artifacts in oral and maxillofacial of Discovery spectral CT, but the performance of Revolution CT combined with multi-material artifact reduction (MMAR) in reducing artifacts of metallic implants in the maxillofacial region has not been explored. In the present study, two techniques were employed for image reconstruction of artifact reduction, aiming to compare their performance in artifact reduction and evaluate the advantage of Revolution CT in reducing metallic artifacts.

## Methods

### Clinical information

A total of 60 patients who received CT scanning of artificial dental implants or fillings between May 2018 and April 2019 were enrolled in our hospital. We confirmed patients had dental fillings or implants before scanning, and then they were randomly arranged on a device of two for examination. The patients were divided into two groups (*n* = 30 per group). Revolution CT and Discovery 750HD CT (GE Corporation, USA) were used for scanning. In the Revolution CT group, there were 12 males and 18 females with a mean age of 45.2 ± 9.3 years (range 32–78 years). In the Discovery CT group, there were 17 males and 13 females with a mean age of 47.3 ± 12.6 years (range 29–86 years).

### CT scanning and image processing

Revolution CT and Discovery CT 750 HD (GE Healthcare, Waukesha, WI, USA) were performed separately in the two groups, using fast kV-switching Gemstone spectral imaging (GSI) between 80 kVp and 140 kVp.The Revolution CT scans acquired 0.5 s rotation speed, automatic mA, 0.992:1 pitch, and ASIR-V50% reconstruction algorithm. The Discovery CT scans acquired 0.5 s rotation speed, automatic mA, 1.375:1 pitch, and ASIR 50% reconstruction algorithm. All CT scans were obtained parallelly to the orbitomeatal line. The contrast agent (iohexol or iodophor 350 mgI/ml) was injected at a flow rate of 3 ml/s at 0.8 ml/kg. A scan delay of 25 s (arterial phase) and 50 s (venous phase) was used. The CT dose index (CTDIvol) and dose length product (DLP) of Revolution CT group were 21.5 mGy and 620.9 mGy-cm, the CTDIvol and DLP of Discovery CT group were 27.8 mGy and 822.1 mGy-cm, respectively.

After scanning at the kiloelectronvolt level of 50, 70, 90, 110, and 130, the data of Revolution CT group were reconstructed into VMI + MMAR images, and the data of Discovery CT group were reconstructed into VMI + MARS images, respectively, with a layer thickness of 0.625 mm. These images were then transferred to the Aw4.7 workstation for image analysis with the GSIviewer software.

### Image analysis and measurement

Both quantitative and qualitative analyses were performed by two radiologists with 4 years of experience in maxillofacial imaging. For quantitative analysis, the region of interest (ROI) was drawn in the following regions: soft tissue with the most obvious artifacts ( soft palate, mouth floor), which was considered to be ROI1; soft tissue without artifacts (musculus longus capitis) at the same scan slice, which was considered to be ROI2. ROI was manually drawn elliptical or circle, the standard ROI size was 40–60 mm^2^. Attenuation (HU) and standard deviation were recorded. The contrast to noise ratio (CNR) and artifact index (AI) were calculated as follows to assess the image quality.

$$ \mathrm{CNR}=\mid \mathrm{CT}1-\mathrm{CT}2\mid /\sqrt{\left( SD{1}^2- SD{2}^2\right)/2},\mathrm{AI}=\sqrt{SD{1}^2- SD{2}^2} $$

For qualitative analysis, two authors (neuroradiologists with 7 and 28 years of experience, respectively) assessed the image quality subjectively on 4-point scales as follows: 1 (unacceptable): metal artifacts are massive, the sharpness of the image is poor, and the oral structure and surrounding tissues is nearly unrecognizable; 2 (poor): metal artifacts are pronounced, and the oral structure and surrounding tissues can be identified but blurred; 3 (fair): metal artifacts are moderate, the oral structure is normal, and the surrounding tissues can be distinguished; 4 (good): there are no or only minor streak artifacts, the oral texture is natural, and the surrounding tissues can be clearly distinguished. Any discrepancy between the two radiologists should be resolved by consultation.

### Statistical analysis

The database was established with Microsoft Excel and statistical analysis was performed using SPSS version 17.0. The continuous variables are expressed by *x* ± *s*. The attenuation, SD, CNR, and AI in two groups displayed abnormal distribution and thus Mann-Whitney rank-sum test was employed for the comparison of these variables; Wilcoxon rank-sum test was used to compare the subjective scores of images; Kappa was used to evaluate the consistency between two observers: Kappa < 0.40, poor consistency; 0.40 ≤ Kappa < 0.75, generally consistent; Kappa ≥ 0.75, favorably consistent. A value of *p* < 0.05 was considered statistically significant.

## Results

### Attenuation, SD, CNR, and AI

**Fig. 1:**
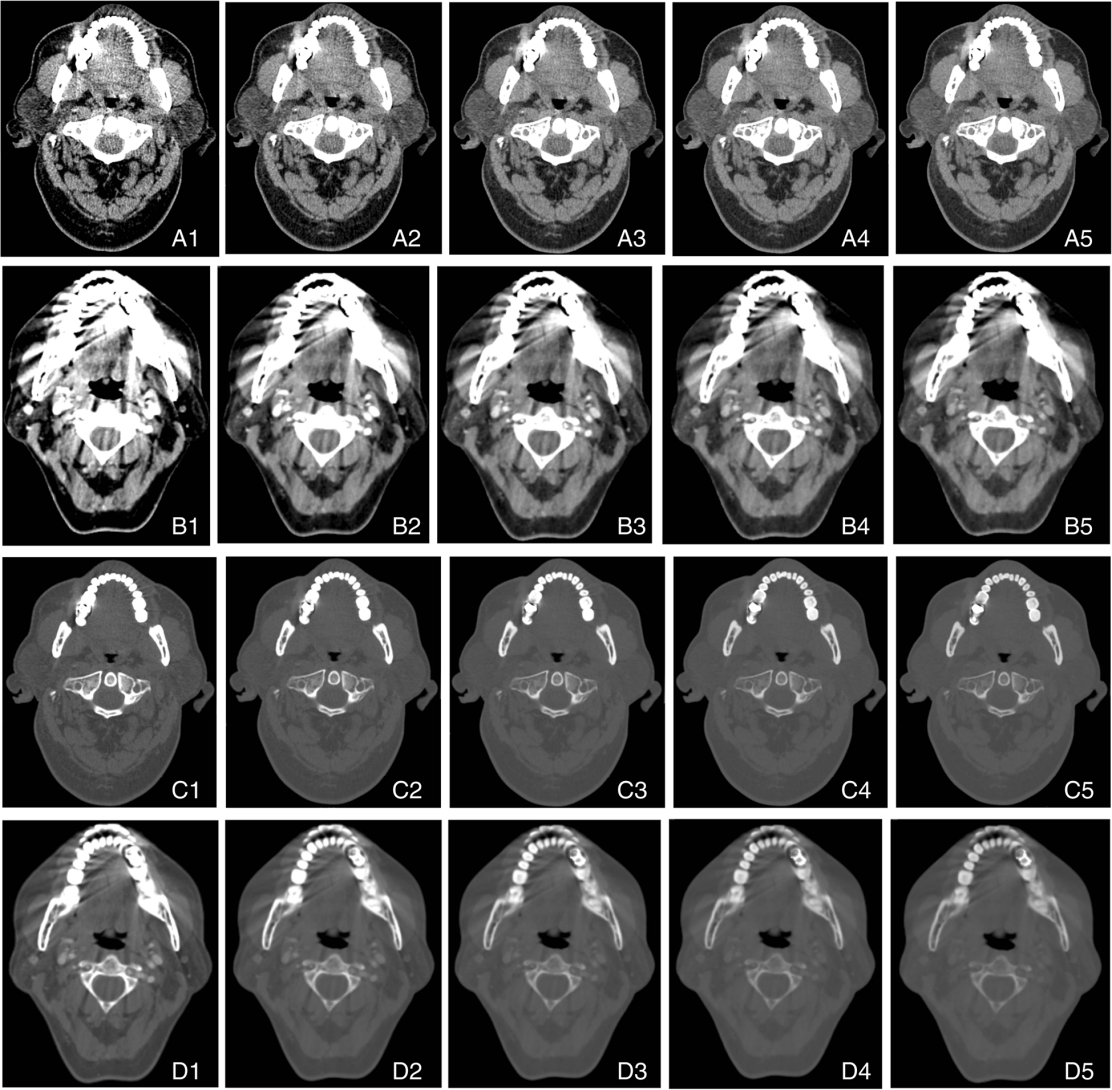
**A1**–**A5** Reconstruction of soft tissue window at VMI_50 keV_, VMI_70 keV_, VMI_90 keV_, VMI_110 keV_, and VMI_130 keV_ in MMAR group. **C1**–**C5** Reconstruction of bone window at VMI_50 keV_, VMI_70 keV_, VMI_90 keV_, VMI_110 keV_, and VMI_130 keV_ in MMAR group. Radial high-density radial artifacts are visible around the metal implants. The artifacts significantly reduce, and the adjacent structures become clear with the increase in the kiloelectronvolt. This suggests the artifact reducing effect is favorable and the implant is clearly distinguishable. **B1**–**B5** Reconstruction of soft tissue window at VMI_50 keV_, VMI_70 keV_, VMI_90 keV_, VMI_110 keV_, and VMI_130 keV_ in MARS group. **D1**–**D5** Reconstruction of bone window at VMI_50 keV_, VMI_70 keV_, VMI_90 keV_, VMI_110 keV_, and VMI_130 keV_ in MARS group. The artifact reducing effect is poor after use of MARS for artifact reduction, and the image quality is not significantly improved; the artifact reducing effect is the best in **B5**, but the contrast of soft tissues at the bottom of the mouth reduces

When the energy level was higher than 70 keV, the attenuation, SD, CNR, and AI in the Revolution CT group were lower than in the Discovery CT group. At VM_I90 keV_ and VMI_110 keV_, the SD, CNR, and AI in the Revolution CT group were significantly lower than in the Discovery CT group (*p* = 0.031, *p* = 0.035, *p* = 0.019, *p* = 0.010, *p* = 0.005, and *p* = 0.008). At VMI_50 keV_, VMI_70 keV_, and VMI_130 keV_, the SD, CNR, and AI were comparable between the two groups (*p* > 0.05). The attenuation was similar between the two groups at any energy level (*p* > 0.05) (Table 1). In the Revolution CT group, the attenuation and CNR were the highest at VMI_50 keV_, the SD and AI were the lowest at VMI_110 keV_ and VMI_130 keV_, and there were no statistically significant differences in the SD and AI between VMI_110 keV_ and V_MI130 keV_ (*p* = 0.283 and *p* = 0.294) (Fig. 1)

**Table 1:** Attenuation, SD, CNR, and AI in two groups at different energy levels (*x* ±*s*, *n* = 30)

	Attenuation		*p*	SD		*p*	CNR		*p*	AI		*p*
	MMAR	MARS		MMAR	MARS		MMAR	MARS		MMAR	MARS	
VMI_50 keV_	192.21 ± 126.27	232.77 ± 186.18	0.375	133.97 ± 58.80	128.50 ± 39.62	0.994	1.66 ± 1.06	2.38 ± 1.79	0.143	131.55 ± 59.61	127.44 ± 38.85	0.859
VMI_70 keV_	100.35 ± 57.85	109.50 ± 95.75	0.525	83.75 ± 28.54	90.60 ± 33.87	0.554	1.21 ± 0.79	1.54 ± 1.08	0.322	82.37 ± 29.07	89.95 ± 34.10	0.501
VMI_90 keV_	64.02 ± 35.14	75.44 ± 81.25	0.455	55.36 ± 18.04	69.73 ± 27.33	0.031	0.91 ± 0.69	1.52 ± 1.20	0.035	54.13 ± 18.68	69.19 ± 27.47	0.019
VMI_110 keV_	46.40 ± 28.12	60.22 ± 73.99	0.287	38.68 ± 12.91	55.12 ± 26.40	0.010	0.96 ± 0.94	1.95 ± 1.67	0.005	37.37 ± 13.66	54.60 ± 26.48	0.008
VMI_130 keV_	36.69± 26.57	50.05 ± 70.17	0.228	29.99 ± 11.01	42.50 ± 25.18	0.071	1.56 ± 1.33	2.44 ± 2.57	0.060	28.72 ± 11.67	41.93 ± 25.21	0.053

.

### Scores of metallic artifacts

The VMI image quality was assessed in two groups. Results showed at VMI_50 keV_, VMI_70 keV_, VMI_90 keV_, VMI_110 keV_, and VMI_130 keV_, the objective score was 2.67 ± 0.48, 3.03 ± 0.49, 3.60 ± 0.50, 3.70 ± 0.47, and 3.73 ± 0.50, respectively, in the Revolution CT group and 1.13 ± 0.35, 1.37 ± 0.56, 2.07 ± 0.69, 2.47 ± 0.57, and 2.73 ± 0.52, respectively, in the Discovery CT group, and the image quality in the Revolution CT group was better than in the Discovery CT group (*P* = 0.001) (Table 2). In addition, the image quality varied significantly with the increase in energy, and the score was the highest at VMI_130 keV_. In the Revolution CT group, scores 3 and 4 were found in 26.7% (8/30) and 73.3% (22/30) of patients. In the Discovery CT group, score 3 was found in 76.7% (23/30) of patients, the image artifacts were not completely removed at any energy level, and score 4 was not found in any image (Table 3). In addition, there were no statistically significant differences in the score at VMI_90 keV_, VMI_110 keV_, and VMI_130 keV_ in the Revolution CT group; or VMI_50 keV_ between VMI_70 keV_ and VMI_110 keV_ between VMI_130 keV_ in the Discovery CT group. The consistency was favorable between two observers (Kappa = 0.764). Both the CTDIvol and DLP of the Revolution CT group were lower than those of the Discovery CT group (*p* = 0.000 and *p* = 0.005).

**Table 2:** Image scores at different energy levels (*x* ± *s*, *n* = 30)

Objective score	MMAR	MARS	*p*
VMI_50 keV_	2.67 ± 0.48	1.13 ± 0.35	0.001
VMI_70 keV_	3.03 ± 0.49	1.37 ± 0.49	0.001
VMI_90 keV_	3.60 ± 0.50	2.07 ± 0.69	0.001
VMI_110 keV_	3.70 ± 0.47	2.50 ± 0.51	0.001
VMI_130 keV_	3.73 ± 0.45	2.73 ± 0.50	0.001

**Table 3:** Image quality at different energy levels

	Group	VMI_50 keV_		VMI_70 keV_		VMI_90 keV_		VMI_110 keV_		VMI_130 keV_	
		MMAR	MARS	MMAR	MARS	MMAR	MARS	MMAR	MARS	MMAR	MARS
Score	1	1	26	0	19	0	6	0	1	0	1
	2	10	4	3	11	0	16	0	14	0	6
	3	19	0	23	0	12	8	9	15	8	23
	4	0	0	4	0	18	0	21	0	22	0
Total		30	30	30	30	30	30	30	30	30	30

As compared to reconstruction with MARS technique, the reconstruction with MMAR technique is better to reduce metallic artifacts and may achieve better image quality. VMI_110 keV_ is the optimal energy level for Revolution CT in acquiring optimal image quality and reducing hardening artifacts.

## Discussion

In this study, we evaluated the virtual monoenergetic image of Revolution CT combined with the MMAR technique, which was compared with the VMI of DECT combined with the MARS technique in reducing metal dental implant artifacts. There have been studies that affirm the VMI and MARS of Discovery CT could remove the metal artifacts effective, but that of Revolution CT and the difference between the two have not been clearly discussed. Our results showed that VMI + MMAR technique led to a decrease of artifacts both, quantitatively and qualitatively, which resulted in visually higher image quality and improved assessment of adjacent soft tissue.

The use of DECT in head and neck imaging has been growing in recent years. The main advantage of DECT is that several additional datasets are obtained without a radiation dose penalty [10]. Improved image quality, better lesion detection, and quantitative calculation of the degree of enhancement are immediate well-recognized benefits [11]. VMI and iodine characterization of DECT may play a major role in patients with head and neck cancer in the detection and delineation of the tumor, resulting in more accurate staging [12], equivalent to the perfusion map of perfusion CT of head and neck cancer [13]. It can differentiate normal, inflammatory, and metastatic squamous cell carcinoma cervical lymph nodes based on iodine concentration [14], as well as between benign post-treatment changes from the primary or recurrent head and neck malignancies [15]. Three material differentiation algorithms for identification of iodine and calcium can be used to assess cartilage and bone marrow infiltration, the latter being a new application in head and neck DECT [16]. Imaging of infection and inflammation can be mitigated with DECT [17], and differential diagnosis can be facilitated with the use of spectral curves. VMI at higher kiloelectronvolt is useful for the reduction of metallic artifacts.

The artifacts of the dental metal prosthesis are star-shaped or radial, which is related to the composition, position, shape, and arrangement of the metal implants [18]. The metal artifacts significantly make it difficult to distinguish important anatomical structures or pathologic conditions, and may even cause the missed diagnosis and misdiagnosis, affecting the correct diagnosis. Conventional CT can reduce tooth artifacts by using thinner slice reconstruction, higher mAs and kVp, and improved reconstruction algorithms [19–22]. The suppression of metal artifacts by these methods is very limited, and there is even the disadvantage of increasing the radiation dose. In this study, two main techniques of the DECT were used to reduce metal artifacts: synthesize monochromatic or monoenergetic imaging and MARS algorithm to remove metallic dental artifacts.

Since the clinical introduction of DECT, this technique has proven to be beneficial in the metal artifact reduction arsenal. Virtual monochromatic imaging (VMI) allows for image reconstruction at different virtual monochromatic energies instead of using a polychromatic spectrum. At higher virtual energies, beam hardening artifacts can be reduced and SNR is increased, because the high energy X-ray still possesses favorable energy uniformity although its energy attenuates after crossing the metal. In addition, high energy X-ray has better penetrating capability, which reduces the energy attenuation when passing through the metal and therefore reduces the photon starvation effect, but photon starvation will still exist and impair the quality of the image [23], the MARS technique, can process signals to provide accurate projection data on the metal implant and its surrounding tissues, which effectively reduces the common metal artifacts and hardening artifacts of other rays [18, 24]. With the combination of the MARS algorithm, the artifact reduction can effectively eliminate the artifacts generated by metal fixtures, implants, metal clips, and spring coils implanted in different parts of the body and can also effectively assess the surrounding tissues [25].

Previous studies on the role of DECT in reducing dental metal implant artifacts have shown that the metal tooth artifacts are significantly reduced in the Discovery CT monochromatic images, which improve the delineation of the metallic prosthesis itself as well as the periprosthetic region. The optimal monoenergetic level for artifacts reduction ranged from 110 to 140 keV [7, 8]. There is evidence showing that DECT combined with MARS can reduce metallic dental artifacts and improve delineation of the metallic prosthesis and periprosthetic region in the buccal area and the tongue, but not in the parotid area; MARS and high energy images are better to display the edge and internal structure of the metal dentures as compared to low energy images; MARS-related artifacts most commonly occurred in the deep center of the neck [9].

Based on the ability of DECT to reduce hardening artifacts by monochromatic imaging, the new generation of 16-cm-wide detector Revolution CT incorporates MMAR technique into the volume reconstruction algorithm [26]. In this technique, different substances are separated in the image reconstruction, aiming to reduce or even eliminate hardening artifacts and improve the image quality.

The objective results suggested the attenuation, SD, and AI of the artifacts decreased with the increase of kiloelectronvolt level. At VMI_90 keV_ and VMI_110 keV_, the SD and AI in the Revolution CT group were lower than those in the Discovery CT group, and the SD and AI were the lowest at VMI_110 keV_ and VMI_130 keV_ in the Revolution CT group, indicating that VMI_110 keV_ was the optimal virtual energy level for Revolution CT in reducing artifacts, with a better image quality, lower image noise, with artifacts, reduced more effectively. Subjective scoring showed that the image quality in the Revolution CT group was better than in the Discovery CT group at any kiloelectronvolt level and the image score increased with the increase of energy level. Although the increasing kiloelectronvolt level would increase the efficient X-ray energy, improve the beam penetration and reduce metal artifact [2], high energy VMI of DECT led to a measurable and visible reduction of artifacts, but the contrast between soft tissues also decreased, thus delineation of soft tissues or contrast-enhancing lesions was more difficult on the high-energy images. The scores of image quality with VMI_130 keV_ were the highest compared to those with VMI_90 keV_, VMI_110 keV_, the difference was not statistically significant in the Revolution CT group. Combined with objective analysis results, we considered VMI_110 keV_ as a reasonably high energy to evaluate metal as well as soft tissues. The radiation dose of the Discovery CT group was consistent with the results of Cha et al. [9], who reported the CTDIvol and DLP were 26.9 mGy and 866.8 mGy-cm, the CTDIvol and DLP of the Revolution CT were 21.5 mGy and 620.9 mGy-cm. Our study found the application of Revolution CT artifact reduction technology not only had better image quality, but also less radiation dose, which is very beneficial for patients.

There were several limitations to this study. First, the exact composition of the dental prostheses was unknown. Because of the retrospective study in nature, before CT scanning, the material and type of the dental prosthesis were unclear. The composition of the prosthesis may affect the image quality to different extents. It has been reported that the performance of MARS is effective for the visualization of stainless steel, but not for titanium [18, 27]. Second, the performance of MMAR in reducing artifacts of soft tissues under different physiological and pathological conditions was not further investigated. For example, whether the performance of MMAR in reducing artifacts in the case of malignant tumors or infectious diseases is different from that in other diseases is still unclear, and further investigation is needed to evaluate the benefit of artifact reduction. Third, the Revolution CT and Discovery CT are different in the artifact reconstruction algorithm, and the iterative reconstruction is not the same between them. The Revolution CT adopts (adaptive statistical iterative reconstruction) ASIR-V, but the Discovery CT uses ASIR for reconstruction although the ratio in the reconstruction algorithm is the same (50%). ASIR-V has the potential to provide image quality equal to or greater than ASIR, with a dose reduction of around 40% [28]. Abdominal CT images reconstructed with ASIR-V facilitate radiation dose reductions of to 35% when compared with the ASIR [29]. In CT portal venography, the application of 80 kV and ASIR-V reconstruction in slender patients can significantly reduce radiation dose (by 63.3%) and contrast agent dose (by 39.7%), compared with the recommended 40% ASIR using 120 kV [30]. For trauma patients, whole-body computed tomography using a low-dose biphasic injection protocol reduced the radiation dose with the maintenance of diagnostic accuracy and image quality after implementing ASiR-V algorism, as compared with routine protocol [31]. Whether the difference algorithm affects the performance of artifact reduction should be considered in further study.

## Conclusions

In conclusion, we found that compared to the 64-slice Discovery CT VMI combined with MARS technique for image reconstruction, the 256-slice Revolution CT combined with MMAR technique for image reconstruction is better to reduce metal artifacts and background SD. The combined use of VMI_110 keV_ + MMAR technique is helpful for observation and evaluation of small structures around the metal implants because the imaging quality is improved.

## Data Availability

The datasets used and/or analyzed during the current study are available from the corresponding author on reasonable request.

## Abbreviations

AI: Artifact index
ASIR: Adaptive statistical iterative reconstruction
CNR: Contrast to noise ratio
DECT: Dual-energy CT
GSI: Gemstone spectral imaging
MARS: Multiple artifact reduction system
MMAR: Multi-material artifact reduction
ROI: Region of interest
SD: Standard deviation

## References

[1] Gong XY, Meyer E, Yu XJ (2013). Clinical evaluation of the normalized metal artefact reduction algorithm caused by dental fillings in CT. Dentomaxillofac Radiol.

[2] De Crop A, Casselman J, Van Hoof T (2015). Analysis of metal artifact reduction tools for dental hardware in CT scans of the oral cavity: kVp, iterative reconstruction, dual-energy CT, metal artifact reduction software: does it make a difference?. Neuroradiology.

[3] Huang ZJ, Liu Y, Xiao ZB, Cao CY, Chen JW (2013) Gemstone CT spectral imaging for metallic artifacts reduction in patients with spine metal implanted: a clinical application study. Chin Comput Med Imag 19:79–83

[4] Bongers MN, Schabel C, Thomas C (2015). Comparison and combination of dual-energy- and iterative-based metal artefact reduction on hip prosthesis and dental implants. PLoS One.

[5] Grosse Hokamp N, Neuhaus V, Abdullayev N (2018). Reduction of artifacts caused by orthopedic hardware in the spine in spectral detector CT examinations using virtual monoenergetic image reconstructions and metal-artifact-reduction algorithms. Skeletal Radiol.

[6] Huang JY, Kerns JR, Nute JL (2015). An evaluation of three commercially available metal artifact reduction methods for CT imaging. Phys Med Biol.

[7] Sun Q, Dong MJ, Yang X, Jiang MD, Tao XF (2017) Clinical analysis of spectrum CT imaging reducing metal artifacts of oral and maxillofacial region. Shanghai Kou Qiang Yi Xue 26:646–649 29691563

[8] Lin X, Wang W, Zhao X (2017). The value of spectral imaging in reducing dental restoration material artifacts. J Clin Radiol.

[9] Cha Jihoon, Kim Hyung-Jin, Kim Sung Tae, Kim Yi Kyung, Kim Ha Youn, Park Gyeong Min (2017). Dual-energy CT with virtual monochromatic images and metal artifact reduction software for reducing metallic dental artifacts. Acta Radiologica.

[10] Tawfik AM, Kerl JM, Razek AA (2011). Image quality and radiation dose of dual-energy CT of the head and neck compared with a standard 120-kVp acquisition. AJNR Am J Neuroradiol.

[11] Vogl Thomas J., Schulz Boris, Bauer Ralf W., Stöver Timo, Sader Robert, Tawfik Ahmed M. (2012). Dual-Energy CT Applications in Head and Neck Imaging. American Journal of Roentgenology.

[12] Roele ED, Timmer VCML, Vaassen LAA, van Kroonenburgh AMJL, Postma AA (2017) Dual-energy CT in head and neck imaging. Curr Radiol Rep 5:19 10.1007/s40134-017-0213-0 PMC5371622 28435761

[13] Razek Ahmed Abdel Khalek Abdel, Tawfik Ahmed Mohamed, Elsorogy Lamiaa Galal Ali, Soliman Nermin Yehia (2014). Perfusion CT of head and neck cancer. European Journal of Radiology.

[14] Tawfik Ahmed M., Razek A. A., Kerl J. Matthias, Nour-Eldin N. E., Bauer Ralf, Vogl Thomas J. (2013). Comparison of dual-energy CT-derived iodine content and iodine overlay of normal, inflammatory and metastatic squamous cell carcinoma cervical lymph nodes. European Radiology.

[15] Yamauchi Hideomi, Buehler Mark, Goodsitt Mitchell M., Keshavarzi Nahid, Srinivasan Ashok (2016). Dual-Energy CT–Based Differentiation of Benign Posttreatment Changes From Primary or Recurrent Malignancy of the Head and Neck: Comparison of Spectral Hounsfield Units at 40 and 70 keV and Iodine Concentration. American Journal of Roentgenology.

[16] Poort Lucas J., Stadler Annika A.R., Ludlage Johan H.B., Hoebers Frank J.P., Kessler Peter A.W. H., Postma Alida A. (2017). Detection of Bone Marrow Edema Pattern With Dual-Energy Computed Tomography of the Pig Mandible Treated With Radiotherapy and Surgery Compared With Magnetic Resonance Imaging. Journal of Computer Assisted Tomography.

[17] Scholtz JE, Husers K, Kaup M (2015). Evaluation of image quality and dose reduction of 80 kVp neck computed tomography in patients with suspected peritonsillar abscess. Clin Radiol.

[18] Lee Young Han, Park Kwan Kyu, Song Ho-Taek, Kim Sungjun, Suh Jin-Suck (2012). Metal artefact reduction in gemstone spectral imaging dual-energy CT with and without metal artefact reduction software. European Radiology.

[19] Bamberg Fabian, Dierks Alexander, Nikolaou Konstantin, Reiser Maximilian F., Becker Christoph R., Johnson Thorsten R. C. (2011). Metal artifact reduction by dual energy computed tomography using monoenergetic extrapolation. European Radiology.

[20] Haramati N, Staron RB, Mazel-Sperling K (1994). CT scans through metal scanning technique versus hardware composition. Comput Med Imaging Graph.

[21] Kotsenas AL, Michalak GJ, DeLone DR (2015). CT metal artifact reduction in the spine: can an iterative reconstruction technique improve visualization?. AJNR Am J Neuroradiol.

[22] Lee MJ, Kim S, Lee SA (2007). Overcoming artifacts from metallic orthopedic implants at high-field-strength MR imaging and multi-detector CT. Radiographics.

[23] Kuchenbecker Stefan, Faby Sebastian, Sawall Stefan, Lell Michael, Kachelrieß Marc (2015). Dual energy CT: How well can pseudo-monochromatic imaging reduce metal artifacts?. Medical Physics.

[24] Verburg JM, Seco J (2012). CT metal artifact reduction method correcting for beam hardening and missing projections. Phys Med Biol.

[25] Brook Olga R., Gourtsoyianni Sofia, Brook Alexander, Mahadevan Anand, Wilcox Carol, Raptopoulos Vassilios (2012). Spectral CT with Metal Artifacts Reduction Software for Improvement of Tumor Visibility in the Vicinity of Gold Fiducial Markers. Radiology.

[26] Chaikriangkrai Kongkiat, Choi Su Yeon, Nabi Faisal, Chang Su Min (2014). Important Advances in Technology and Unique Applications to Cardiovascular Computed Tomography. Methodist DeBakey Cardiovascular Journal.

[27] Douglas-Akinwande Annette C., Buckwalter Kenneth A., Rydberg Jonas, Rankin James L., Choplin Robert H. (2006). Multichannel CT: Evaluating the Spine in Postoperative Patients with Orthopedic Hardware. RadioGraphics.

[28] Gatti M, Marchisio F, Fronda M (2018). Adaptive statistical iterative reconstruction-V versus adaptive statistical iterative reconstruction: impact on dose reduction and image quality in body computed tomography. J Comput Assist Tomogr.

[29] Ren Zhanli, Zhang Xirong, Hu Zhijun, Li Dou, Liu Zhentang, Wei Donghong, Jia Yongjun, Yu Nan, Yu Yong, Lei Yuxin, Chen Xiaoxia, Guo Changyi, Ren Zhanliang, He Taiping (2020). Reducing Radiation Dose and Improving Image Quality in CT Portal Venography Using 80 kV and Adaptive Statistical Iterative Reconstruction-V in Slender Patients. Academic Radiology.

[30] Kwon H, Cho J, Oh J (2015). The adaptive statistical iterative reconstruction-V technique for radiation dose reduction in abdominal CT: comparison with the adaptive statistical iterative reconstruction technique. Br J Radiol.

[31] Elmokadem Ali H., Ibrahim Enas A., Gouda Walaa A., Khalek Abdel Razek Ahmed Abdel (2019). Whole-Body Computed Tomography Using Low-Dose Biphasic Injection Protocol With Adaptive Statistical Iterative Reconstruction V. Journal of Computer Assisted Tomography.

